# Cerebrospinal Fluid Findings in an Adult with Human Metapneumovirus–Associated Encephalitis

**DOI:** 10.3201/eid2302.161337

**Published:** 2017-02

**Authors:** Natalie Jeannet, Bernadette G. van den Hoogen, Joerg C. Schefold, Franziska Suter-Riniker, Rami Sommerstein

**Affiliations:** University of Bern, Bern, Switzerland (N. Jeannet, J.C. Schefold, F. Suter-Riniker, R. Sommerstein);; Erasmus Medical Center, Rotterdam, the Netherlands (B.G. van den Hoogen);; Bern University Hospital, Bern (J.C. Schefold, R. Sommerstein)

**Keywords:** meningitis/encephalitis, viruses, human metapneumovirus, antibodies, laboratory diagnosis, encephalitis, the Netherlands, IgG, IgM, immunofluorescence, cerebrospinal fluid

**To the Editor:** Acute encephalitis/encephalopathy associated with human metapneumovirus (HMPV) has been documented in children (*1*–*3*). Recently, Fok et al. (*4*) described an encephalitis case in an adult but were unable to test cerebrospinal fluid (CSF) for HMPV. Following authors’ recommendations, we performed diagnostic testing on the CSF of an adult with HMPV-associated encephalitis.

A previously healthy 61-year-old man came to our institution with headache and seizures 5 days after onset of an influenza-like illness. A lumbar puncture on admission revealed pleocytosis (36 cells/µL) and a mononuclear predominance of 98%. Results of magnetic resonance imaging and computed tomography of the head and chest radiography on admission were inconclusive. The patient was treated in the intensive care unit for possible viral and bacterial meningoencephalitis. Although results of routine CSF-workup for infectious causes were unremarkable, total CSF protein level was elevated at 1.39 g/L (reference range 0.2–0.4 g/L). A nasopharyngeal swab specimen was positive for HMPV (cycle threshold 28.6) using duplex reverse transcription PCR (r-gene; Biomérieux, Marcy l’Etoile, France).

However, HMPV reverse transcription PCR results were negative in the concurrent CSF sample. Immunofluorescence assays demonstrated HMPV IgG (serum titer 1:8,192; CSF titers 1:64 and 1:32). Indices calculated using the formula (IgG_CSF_ HMPV/IgG_Serum_ HMPV)/(IgG_CSF_ total/IgG_Serum_ total) were lower than the cut-off value of 4, indicating absence of intrathecal IgG against HMPV (Table). 

**Table:** Results of PCR and immunofluorescent assay testing in adult patient with HMPV*

Test	Sample type, result or IgG titer		HMPV IgG index
	Nasopharyngeal swab	CSF	
Reverse transcription PCR	Positive (cycle threshold 28.6)	Negative	
Immunofluorescence assays			
Vero-118 cells infected with HMPV NL/1/00	1:8,192	1:32	0.54
Vero-118 cells infected with HMPV NL/1/99	1:8,192	1:64	1.07

*A duplex reverse transcription PCR (r-gene) for detection of human metapneumovirus (HMPV) was performed from a nasopharyngeal swab specimen and cerebrospinal fluid (CSF). For immunofluorescence assays, 96-well plates coated with Vero 118 cells were infected with HMPV NL/1/00 and NL/1/99, respectively. Twenty-four hours later, infected cells were incubated with serial dilutions of patient serum and CSF for 1 h at 37°C. After washing with phosphate-buffered saline, plates were incubated with anti-human IgG conjugated with fluorescein isothiocyanate for 1 h at 37°C. Lowest dilution giving a positive result was determined by UV microscopy. Intrathecal IgG synthesis was calculated using the formula (IgG_CSF_ HMPV/IgG_Serum_ HMPV)/(IgG_CSF_ total/IgG_Serum_ total). Indices below 4 indicate absence of intrathecal IgG antibody synthesis.

As in the study by Fok et al. (*4*), our case supports consideration of HMPV as a causative agent of acute encephalitis after respiratory tract infection in adults. We could not demonstrate direct or indirect evidence of HMPV CSF invasion as the cause for HMPV-associated encephalitis in an adult, in contrast to a case in a child in which detection of HMPV in CSF suggested a causative role in acute encephalitis (*1*). Our data may point toward the role of nonspecific inflammatory response as the main pathogenic factor in HMPV-related encephalitis in adults.

## References

[1] Sánchez Fernández I, Rebollo Polo M, Muñoz-Almagro C, Monfort Carretero L, Fernández Ureña S, Rueda Muñoz A, et al. Human metapneumovirus in the cerebrospinal fluid of a patient with acute encephalitis. Arch Neurol. 2012;69:649–52. 10.1001/archneurol.2011.1094 22232205

[2] Vehapoglu A, Turel O, Uygur Sahin T, Kutlu NO, Iscan A. Clinical significance of human metapneumovirus in refractory status epilepticus and encephalitis: case report and review of the literature. Case Rep Neurol Med. 2015;2015:131780. 10.1155/2015/131780 26664779 PMC4667075

[3] Niizuma T, Okumura A, Kinoshita K, Shimizu T. Acute encephalopathy associated with human metapneumovirus infection. Jpn J Infect Dis. 2014;67:213–5. 10.7883/yoken.67.213 24858612

[4] Fok A, Mateevici C, Lin B, Chandra RV, Chong VH. Encephalitis-associated human metapneumovirus pneumonia in adult, Australia. Emerg Infect Dis. 2015;21:2074–6. 10.3201/eid2111.150608 26488420 PMC4622250

